# Sighting acute myocardial infarction through platelet gene expression

**DOI:** 10.1038/s41598-019-56047-0

**Published:** 2019-12-20

**Authors:** Giuliana Gobbi, Cecilia Carubbi, Guidantonio Malagoli Tagliazucchi, Elena Masselli, Prisco Mirandola, Filippo Pigazzani, Antonio Crocamo, Maria Francesca Notarangelo, Sergio Suma, Elvezia Paraboschi, Giuseppe Maglietta, Srikanth Nagalla, Giulia Pozzi, Daniela Galli, Mauro Vaccarezza, Paolo Fortina, Sankar Addya, Adam Ertel, Paul Bray, Stefano Duga, Carlo Berzuini, Marco Vitale, Diego Ardissino

**Affiliations:** 1 Department of Medicine and Surgery, University of Parma, Parma, Italy; 2 Division of Cardiology, Azienda Ospedaliero-Universitaria di Parma, Parma, Italy; 3 Division of Research and Innovation, Azienda Ospedaliero-Universitaria di Parma, Parma, Italy; 4 Department of Biomedical Sciences, Humanitas University, Milan, Italy; 5 Department of Statistics, Computer science, Applications, Univeristà di Firenze, Florence, Italy; 6 Department of Internal Medicine, UT Southwestern Medical Center, Dallas, TX USA; 7 School of Pharmacy and Biomedical Sciences, Faculty of Health Science, Curtin University, Perth, WA Australia; 8 Sidney Kimmel Cancer center, Department of Cancer Biology, Thomas Jefferson University, Philadelphia, PA USA; 9 Department of Molecula Medicine, Sapienza University, Rome, Italy; 10 Division of Hematology and Hematologic Malignancies, Department of Medicine, University of Utah, Salt Lake City, UT USA; 11 Center for Biostatistics, Institute of Population Health, University of Manchester, Manchester, UK

**Keywords:** Cardiovascular biology, Cardiovascular diseases

## Abstract

Acute myocardial infarction is primarily due to coronary atherosclerotic plaque rupture and subsequent thrombus formation. Platelets play a key role in the genesis and progression of both atherosclerosis and thrombosis. Since platelets are anuclear cells that inherit their mRNA from megakaryocyte precursors and maintain it unchanged during their life span, gene expression profiling at the time of an acute myocardial infarction provides information concerning the platelet gene expression preceding the coronary event. In ST-segment elevation myocardial infarction (STEMI), a gene-by-gene analysis of the platelet gene expression identified five differentially expressed genes: FKBP5, S100P, SAMSN1, CLEC4E and S100A12. The logistic regression model used to combine the gene expression in a STEMI vs healthy donors score showed an AUC of 0.95. The same five differentially expressed genes were externally validated using platelet gene expression data from patients with coronary atherosclerosis but without thrombosis. Platelet gene expression profile highlights five genes able to identify STEMI patients and to discriminate them in the background of atherosclerosis. Consequently, early signals of an imminent acute myocardial infarction are likely to be found by platelet gene expression profiling before the infarction occurs.

## Introduction

Acute myocardial infarction is a sudden event that is fatal in approximately one-third of patients, with about half of the deaths occurring within one hour. It is frequently attributed to coronary atherosclerotic plaque rupture and the consequent exposure of thrombogenic substances that promote platelet activation/aggregation, the activation of coagulation and, ultimately, the formation of an intraluminal thrombus that limits coronary blood flow^1,2^. However, although plaque rupture or fissuring frequently occurs in atherosclerosis, only a small proportion of ruptured plaques results in thrombosis. Autopsy data show that a sizable proportion of the subjects who die suddenly of non-cardiac causes have fissured plaques without thrombosis in their coronary arteries^3^. The rupture of coronary atherosclerotic plaque is therefore much more frequent than thrombus formation. It has been suggested that individual reactivity to plaque rupture may play a causative role in provoking the clinical event of acute myocardial infarction^4–8^.

Platelets are key mediators of hemostasis that contribute to the genesis and progression of atherosclerosis, and the precipitation of an atherothrombotic event after plaque rupture^1,2,9^. Platelets are anuclear cells formed from the cytoplasm of megakaryocytes (MKs) and circulate in the blood stream for about 7–10 days^10^. Despite the absence of a nucleus, platelets contain RNAs, including messenger RNA (mRNA), long non-coding RNA and micro-RNA, and protein synthesis machinery accounting for more than 9,000 transcripts in healthy human donors^11–13^. Platelet mRNA is primarily derived from MKs: indeed, genome-wide expression studies of platelets and MK-derived cultures from CD34 + hematopoietic progenitor cells, have shown that about half of the approximately 10,000 transcripts present in MKs can also be found in platelets^14,15^.

Platelet transcriptome alterations have been associated with specific human phenotypes such as coronary artery disease^16–18^, atrial fibrillation^19^, essential thrombocythemia^20^, systemic lupus erythematosus^21^ and sickle cell anemia^22^. The transcriptional profiling of platelets provides information concerning the gene expression that precedes an acute coronary event without the confounding possibility of *de novo* gene transcription. This study was therefore designed to test the hypothesis that platelet transcriptome contains the gene signature of an imminent myocardial infarction.

## Results

**Figure 1:**
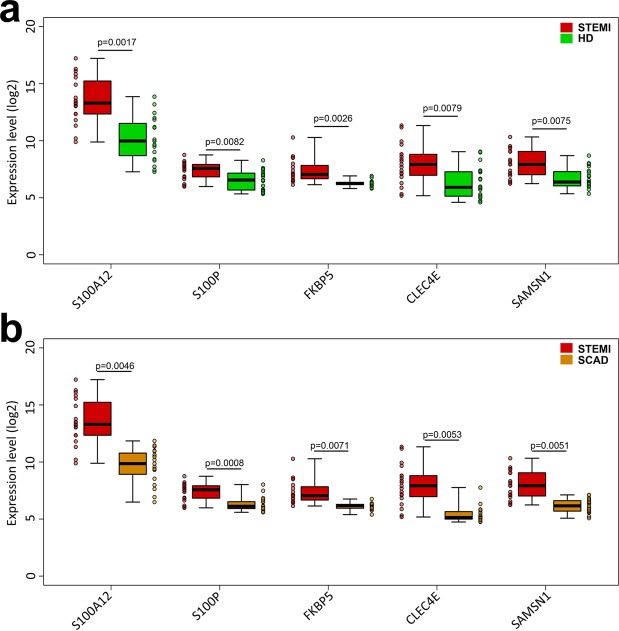
Gene expression values of the five identified genes. Panel a: Individual gene expression values in STEMI patients (red), and healthy subjects (green) with median values and interquartile ranges of the five identified genes. Panel b: Individual gene expression values in STEMI patients (red), and SCAD patients (orange) with median values and interquartile ranges of the five identified genes.

**Figure 2:**
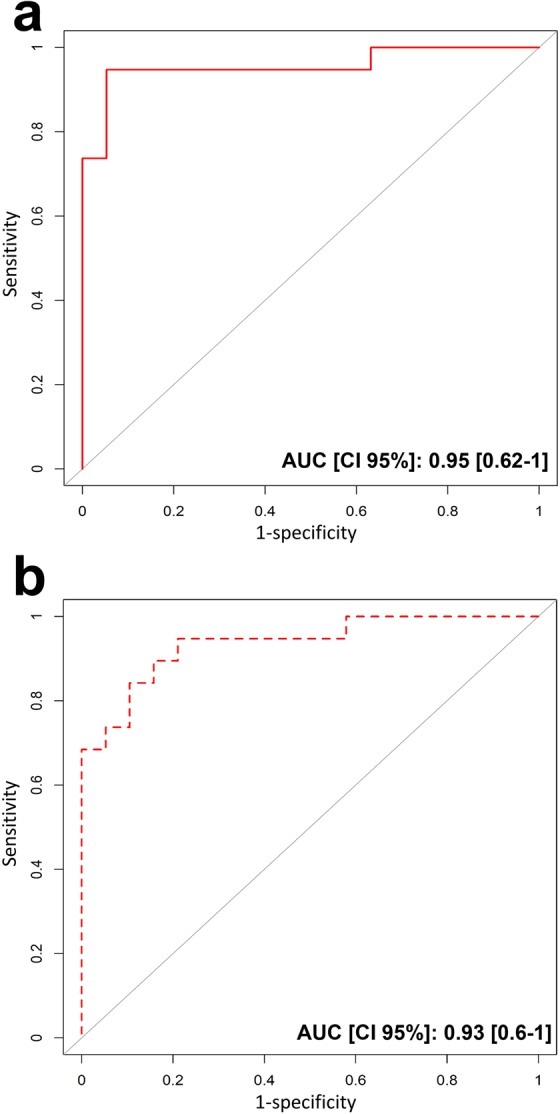
Receiver operating characteristic (ROC) curve for discriminating STEMI from HD and from SCAD. (**a**) The area under the ROC curve (AUC) of the capacity of the five identified genes to discriminate STEMI patients and HD (red line). AUC of 0.95 (95% confidence interval 0.62–1.00). (**b**) The area under the ROC curve (AUC) of the capacity of the five identified genes to discriminate STEMI and SCAD patients (red dotted line). AUC of 0.93 (95% confidence interval 0.60–1.00).

The gene that showed the strongest evidence of differential expression between the ST-segment elevation myocardial infarction (STEMI) and healthy donors (HD) groups (Table 1) was FKBP5 (odds ratio: 204.06; 95% confidence interval 12.11–15705.95; p = 0.0026), followed by S100P (odds ratio: 3.35; 95% confidence interval 1.48–9.25; p = 0.0082), SAMSN1 (odds ratio: 2.76; 95% confidence interval 1.42–6.53; p = 0.0075), S100A12 (odds ratio: 2.35; 95% confidence interval 1.51–4.52; p = 0.0017) and CLEC4E (odds ratio:1.97; 95% confidence interval 1.26–3.47; p = 0.0079) (Fig. 1, panel a and Supplemental Table 1). A logistic regression model based on the five genes was capable of discriminating the STEMI patients from HDs with an area under the curve (AUC) of 0.95 (95% confidence interval 0.62–1.00) (Fig. 2, panel a). These results were internally validated by means of a bootstrap analysis that took gene selection into account. Additionally, we found that the expression levels of these five genes were independent from the time of blood withdrawn (Supplemental Fig. 1). Moreover, we tested the correlation levels between the expression of each of the five identified genes and CK-MB and Troponin I circulating levels. No statistically significant correlation was found except for S100P vs CK-MB (p = 0.56, p = 0.0120) and S100P vs Troponin I (p = 0.5700, p = 0.0110) (Supplemental Fig. 2), in agreement with a previous study on a rat model with ischemia-reperfusion injury^23^.

**Table 1:** Clinical, electrocardiographic, and angiographic variables of the patients.

	STEMI	HD	SCAD
Number of patients, n	20	20	20
Age, median (range)	65.5 (51–82)	62.5 (49–81)	63.5 (51–80)
**Gender**			
*Male, n (%)*	15 (75)	15 (75)	15 (75)
*Female, n (%)*	5 (25)	5 (25)	5 (25)
**Family history**			
*n (%)*	7 (35)	0 (0)	3 (15)
**Hypertension**			
*n (%)*	11 (55)	0 (0)	17 (85)
**Smoking**			
*n (%)*	10 (50)	0 (0)	3 (15)
**Dyslipidemia**			
*n (%)*	9 (45)	0 (0)	11 (55)
**Diabetes Mellitus**			
*n (%)*	5 (25)	0 (0)	7 (35)
**Obesity**			
*n (%)*	2 (10)	0 (0)	3 (15)
**ST elevation**			
*Inferior- lateral, n (%)*	3 (15)	no	no
*Anterior, n (%)*	9 (45)	no	no
*Inferior, n (%)*	7 (35)	no	no
*Lateral, n (%)*	1 (5)	no	no
**Angiography**			
*CX occlusion, n (%)*	4 (20)	n/a	1 (5)
*LAD occlusion, n (%)*	9 (45)	n/a	10
*RCA occlusion, n (%)*	7 (35)	n/a	1
*CX, LAD*	0	n/a	2
*CX, RCA*	0	n/a	1
*LAD, RCA*	0	n/a	1
*CX, LAD, RCA*	0	n/a	4
**Thrombosis**			
*n (%)*	20 (100)	n/a	no
CK-MB, mean ± SD	172.39 ± 95.30	normal	normal
Tn I, mean ± SD	48.44 ± 28.32	normal	normal
**Aspirin**			
*n (%)*	6 (30)	0 (0)	20 (100)
**P2Y12 Inhibitors**			
*n (%)*	0 (0)	0 (0)	0 (0)
**Anticoagulants**			
*n (%)*	0 (0)	0 (0)	0 (0)

Family history of ischemic heart disease in a first-degree relative before 55 years in men and before 65 years in women; ST elevation: location of ST elevation; CX: circumflex coronary artery; LAD: left anterior descending coronary artery; RCA: right coronary artery; Thrombosis: presence of coronary thrombosis at angiography; CK-MB: CK-MB peak in ng/mL; Tn-I: Troponin I peak in ng/ml. Aspirin: patients on treatment with aspirin; P2Y12 Inhibitors: patients on treatment with P2Y12 inhibitors; Anticoagulants: patients on treatment with anticoagulants n: number of patients. N/A: not applicable.

The ability of the five identified genes to discriminate subjects with enhanced reactivity to plaque rupture was externally confirmed using data from a further 20 patients with stable coronary artery disease (SCAD), the phenotypically closest condition to STEMI (Fig. 1, panel b). The logistic model based on these five genes was capable of discriminating the STEMI and SCAD groups with an AUC of 0.93 (95% confidence interval 0.60–1.00) (Fig. 2, panel b).

## Discussion

Here, we first report five differentially expressed genes (DEGs) on platelet transcriptome able to identify STEMI patients from HD. It is worth noting that all 5 DEGs were already known to be associated with cardiovascular conditions: S100A12 with coronary artery disease (CAD) and atherosclerotic plaques^24^; FKBP5 with STEMI^25^; CLEC4E and SAMSN1 with atherosclerotic lesions^26,27^; and S100P with acute coronary syndrome (ACS)^23^ (Table 2). Overall, analysis of platelet gene expression reveals quantitative differences that immediately precede the occurrence of an acute myocardial infarction.Indeed, our data and the existing literature^16,23,24^ strongly suggested that S100 protein family^28^ is a relevant player in cardiovascular risk^29^ and thrombus formation both in mouse^30^ and ACS patients^31^. However, S100 protein family demonstrated a poor perfomance as a biomarker in emergency settings^32,33^. On the contrary, this panel of 5 DEGs allowed a successful sorting of cardiovascular events.

**Table 2:** Functional annotation of the five identified genes.

Gene name	Gene function	Implications in cardiovascular disease	References
***S100A12*** (S100 calcium binding protein A12)	Involved in regulating a number of cell processes (such as cell cycle progression and differentiation), and specific calcium-dependent signal transduction pathways.	Significantly higher S100A12 levels are found in the serum of patients with coronary artery disease than in controls, and correlate positively with C-reactive protein levels.	^24^
***FKBP5*** (FK506 binding protein 5)	A member of the immunophilin protein family, it plays a role in immunoregulation and basic cell processes involving protein folding and trafficking.	More frequent in STEMI than NSTEMI platelets in an RNA-seq analysis of platelet transcriptome in patients with acute myocardial infarction.	^25^
***CLEC4E*** (C-type lectin domain family 4 member E)	Involved in cell adhesion, cell-cell signalling, glycoprotein turnover, inflammation and immune response.	CLEC4E is expressed in human and mouse atherosclerotic lesions and is activated by necrotic lesion extracts. It plays a critical role in promoting a pro-atherogenic macrophage phenotype and aggravates atherosclerotic lesion inflammation.	^26^
***SAMSN1*** (SAM domain, SH3 domain and nuclear localisation signals 1)	A member of a novel gene family of putative adaptors and scaffold proteins. It is a negative regulator of B-cell activation and down-regulates cell proliferation. It promotes membrane ruffle formation and the reorganisation of the actin cytoskeleton.	SAMSN1 expression is up-regulated in B cell activation signalling cascades and in the peripheral blood mononuclear cells and atherosclerotic lesions of femoral arteries in cases of peripheral artery disease.	^27^
***S100P*** (S100 calcium binding protein P)	The protein encoded by this gene is a member of the S100 family of proteins, localised in the cytoplasm and/or nucleus of a wide range of cells and is involved in regulating a number of cell processes such as cell cycle progression and differentiation. In addition to binding Ca^2+^, this protein also binds Zn^2+^ and Mg^2+^.	Serum levels of S100B, S100A6 and S100P are higher in patients with acute coronary syndrome than in patients with stable angina or control subjects. The expression of these proteins is related to myocardial injury in patients with acute coronary syndrome and in rat models of myocardial infarction.	^23^

Functional annotation of the five genes identified in the STEMI *vs* HD analysis with a p-value of < 0.01.

In fact, the simultaneous up-regulation of these five genes, which are known simultaneous up-regulation of these five genes, which are known to be associated with cardiovascular conditions, identified STEMI patients and, in a background of coronary atherosclerosis, discriminated those with or without thrombosis. A limitation of our study is the relatively small sample size, and we are aware that our five genes set has to be validated in larger population cohorts of STEMI subjects and in cohorts of subjects with different degrees of cardiovascular risk. We are also aware that our healthy donor controls could not be the best comparator due to confounding by medications. Non-ACS chronic CAD patients matched for age and medications might be a better comparator to further confirm our results. However, it is our opinion that the stringent inclusion criteria (i.e. blood samples processing within 6 hours; absence of pharmacological treatments before symptoms onset and sample collection) as well as exclusion criteria (as reported in the Methods Section, we excluded all the STEMI patients under anti-thrombotic/anti-coagulant treatment and/or anti-platelet drugs [except aspirin, which is mandatory based on current guidelines] before the time of hospital admission) adopted generated the solid evidence reported. Even if data from the literature reported that aspirin could affect mRNA and protein expression of megakaryocytes and platelets^34–36^, our results unequivocally distinguished STEMI patients from healthy donors (all without aspirin treatment) and from SCAD patients (all on aspirin treatment). Therefore, we believe that aspirin did not interfere with the concurrent differential expression of the five identified genes.

This is the first study demonstrating that platelets gene expression contains useful information to predict an unexpected clinical event such as MI. So far, no other biomarkers proved to be able to predict plaque rupture, the underlying mechanism of acute MI. The logical next step will be a prospective clinical validation in a larger population at high risk for MI and moreover, future studies are also needed to clarify whether the five identified genes have causative role or are simply markers of the acute event to happen. Notwithstanding these caveats, the translational relevance of our findings pre-dating myocardial infarction is evident *per se* and might well represent a valuable predictor for this still unpredictable disease and shed a light into the biological underpinning of acute myocardial infarction that precede current diagnostic tools and potentially lead to changes in the way we approach patients with suspected heart attack in the future.

## Methods

### Patient selection

Three groups of subjects were studied: (1) twenty patients with ST elevation myocardial infarction (STEMI); (2) twenty healthy donor (HD); (3) twenty patient with stable coronary artery disease (SCAD).

All subjects were enrolled at the Cardiology Division of the Azienda Ospedaliero-Universitaria of Parma. This study was performed according to the Declaration of Helsinki, the protocol was approved by the Ethical Committee of Parma University Hospital and subjects were enrolled after written informed consent.

Between June 2014 and October 2015, we prospectively enrolled 20 patients with ST elevation myocardial infarction (STEMI) defined as typical chest pain with the following electrocardiographic criteria: a new ST segment elevation at the J point in two contiguous leads, with cut-off points of >0.1 mV in all leads other than leads V2-V3, for which the cut-off points were ≥0.2 mV in men aged ≥40 years, ≥0.25 mV in men aged <40 years, or ≥0.15 mV in women^37^. Before coronary angiography, all of the patients underwent arterial blood sampling (50 mL) within one our from the hospital admission. For all STEMI patients, the time to chest pain/symptoms onset ranged from 1 to 6 hours before sample collection. The samples were collected before the administration of any anticoagulant or antiplatelet drugs (except acetylsalicylic acid given before hospital arrival). All blood samples have been processed within one hour of collection.

Each STEMI patient (Supplemental Table 2) was sex- and age-matched (±3 years) with a healthy donor (HD) (Supplemental Table 3) and a patient with stable coronary artery disease (SCAD) (Supplemental Table 4).

The HDs were defined as subjects without a previous history of cardiovascular diseases and without any risk factors for ischemic heart diseases. All the HD samples collection were planned within one-hour from medical examination. The HDs underwent venous blood sampling (50 mL), and all of the samples were processed within one hour of collection. The HDs were not taking any drugs (Supplemental Table 3).

SCAD was defined as a documented coronary artery stenosis of >70% in at least one epicardial main artery and documented inducible ischemia and/or symptoms without any characteristic of unstable disease^38^ (i.e. troponin elevation, rest angina, new-onset angina, recent onset of moderate-to-severe angina, or crescendo angina). All the SCAD samples collection were planned within one-hour from medical examination. The SCAD patients underwent venous blood sampling (50 mL), and all of the samples were processed within one hour of collection. At the time of sampling, all of the patients with SCAD were on aspirin and none of them were taking a P2Y12 inhibitor or anticoagulant (Supplemental Table 4).

Patients characteristics and their cardiovascular risk factors are reported in Table 1.

### Sample source and platelet purification

Citrate anti-coagulated blood samples (50 mL) were taken from each subject and collected in vacutainers (BD Vacutainer, Becton Dickinson, San Diego, CA) at a final sodium citrate concentration of 3.8%.

Leukocyte depletion was used to obtain highly purified platelets, as previously described^39,40^. The leucocytes-depleted platelets were treated with an appropriate amount of TRIzol^TM^ (Invitrogen) for cell lysis and RNA cryopreservation.

### Quantitative real-time polymerase chain reaction (PCR)

RNA was extracted from leucocytes-depleted platelets using TRIzol^TM^ (Invitrogen), as previously described^39^. The RNA was reverse-transcribed and semi-quantitative real-time RT-PCRs were performed to detect the expression levels of CLEC4E, FKBP5, SAMSN1, S100A12 and S100P using the SYBR Premix Ex Taq II (Takara, Shiga, Japan) and a LightCycler 480 (Roche, Basel, Switzerland). ACTB (Actin Beta) and ITGA2B (Integrin, Alpha 2b, Platelet Glycoprotein IIb of IIb/IIIa Complex, Antigen CD41) were used as housekeeping genes; the reactions were performed in triplicate, and the expression data were analysed and rescaled using GeNorm software^41^ (Supplemental Fig. 3).

### Microarray hybridisation

RNA was extracted using TRIzol^TM^ (Invitrogen) in accordance with the manufacturer’s protocol, quantified on a Nanodrop ND-100 spectrophotometer, and quality assessed by means of analysis on an Agilent 2200 TapeStation (Agilent Tehnologies, Palo Alto, CA). Fragmented biotin-labelled cDNA was synthesised using the GeneChip WT Pico kit (Affymetrix, Santa Clara, CA). Affymetrix gene chips (Human Transcriptome Array 2.0, Affymetrix, Santa Clara, CA) were hybridised with fragmented and biotin-labelled cDNA. The chips were scanned by an Affymetrix Gene Chip Scanner 3000 using Command Console Software. The experiment was quality controlled using Expression Console Software v 1.4.1.

### Microarray data processing

The expression data obtained from the Affymetrix probe-sets (70,523) were processed using Expression Console software (Affymetrix, www.affymetrix.com) with the default parameters. The data are available at GEO, Accession No. GSE109048

### Statistical analysis

The expression levels of the 10% most variably expressed genes^42^ were collected into a 38 × 6,754 matrix whose rows corresponded to the individual samples and columns corresponded to the selected genes, plus a column for individual STEMI-HD status. Simple logistic regression models^43^ were used to test each of the 6,753 expression variables for any association with the STEMI *vs* HD indicator. There were 51 gene expression variables with a p-value of <0.05 (Supplemental Table 4) and five with a p-value of <0.01. The later were considered for the subsequent stage of analysis, in which they acted as explanatory variables in a multiple logistic regression model for the log-odds ratio of the risk of STEMI *vs* HD. The model was internally validated by means of bootstrap resampling^44,45^. We then ran 30,000 iterations of a bootstrap procedure, each iteration involving resampling with the replacement of the rows of the original data matrix in such a way as to preserve the original matched structure, and analysed the simulated dataset using the same procedure as that applied to the original dataset. The 30,000 bootstrapped Area Under curve (AUC) statistics were used to calculate an empirical mean value and 95% confidence interval for the “true” AUC value. Finally, our logistic predictor of the risk of STEMI *vs* HD was assessed in terms of its ability to discriminate STEMI from SCAD as an external validation of the model. This involved the use of data from further patients with SCAD – the phenotypically closest condition to STEMI with documented coronary atherosclerosis but without thrombosis (Fig. 1, panel b). In this analysis, the logistic predictor was used with the gene-specific coefficients fixed at the values estimated on the basis of the STEMI *vs* HD analysis. The analyses were made using custom R-scripts and R-3.3.2^46^.

### Reticulated platelet analysis

Aliquots of whole blood were stained with thiazole orange (TO) and analysed by means of flow cytometry in order to identify reticulated platelets^47–49^, as previously described^40^ (Supplemental Fig. 4).

For detailed methods see “Supplemental Material”.

### Ethical approval and informed consent statement

The authors stated that this study was performed according to the Declaration of Helsinki, all experimental protocols were approved by the Ethical Committee of Parma University Hospital. The authors confirmed that informed consent was obtained from all participants.

## Supplementary information

Supplementary Information

## Data Availability

The authors stated that the datasets generated by microarray analyses of the current study are available at GEO, Accession No. GSE109048.
