# Supplementary material for: Sighting acute myocardial infarction through platelet gene expression
Source: Sci Rep. 2019 Dec 20;9:19574. doi: 10.1038/s41598-019-56047-0 (PMC6925116; doi:10.1038/s41598-019-56047-0)
Supplement: Supplementary file 1 — Supplementary Information [file 41598_2019_56047_MOESM1_ESM.pdf]

SUPPLEMENTARY INFORMATION

Sighting acute myocardial infarction through platelet gene expression

Giuliana Gobbi, Cecilia Carubbi, Guidantonio Malagoli Tagliazucchi, Elena Masselli, Prisco Mirandola, Filippo Pigazzani, Antonio Crocamo, Maria Francesca Notarangelo, Sergio Suma, Elvezia Paraboschi, Giuseppe Maglietta, Srikanth Nagalla, Giulia Pozzi, Daniela Galli, Mauro Vaccarezza, Paolo Fortina, Sankar Addya, Adam Ertel, Paul Bray, Stefano Duga, Carlo Berzuini, Marco Vitale, Diego Ardissino.

1. Detailed Methods.

Patient selection

Between June 2014 and October 2015, we prospectively enrolled 20 patients with ST elevation myocardial infarction (STEMI) defined as typical chest pain with the following electrocardiographic criteria: a new ST segment elevation at the J point in two contiguous leads, with cut-off points of >0.1 mV in all leads other than leads V2-V3, for which the cut-off points were ≥0.2 mV in men aged ≥40 years, ≥0.25 mV in men aged <40 years, or ≥0.15 mV in women. [1] Before coronary angiography, all of the patients underwent arterial blood sampling (50 mL) within six hours of the onset of chest pain/symptoms. The samples were collected before the administration of any anticoagulant or antiplatelet drugs (except acetylsalicylic acid given before hospital arrival), and processed within one hour of collection.

Each STEMI patient (Supplemental Table 2) was sex- and age-matched (±3 years) with a healthy donor (HD) (Supplemental Table 3) and a patient with stable coronary artery disease (SCAD) (Supplemental Table 4). The HDs were defined as subjects without a previous history of cardiovascular diseases and without any risk factors for ischemic heart diseases. SCAD was defined as a documented coronary artery stenosis of >70% in at least one epicardial main artery and documented inducible ischemia and/or symptoms without any characteristic of unstable disease[2] (i.e. troponin elevation, rest angina, new-onset angina, recent onset of moderate-to-severe angina, or crescendo angina) The SCAD patients and HDs both underwent venous blood sampling (50 mL), and all of the samples were processed within one hour of collection. At the time of sampling, all of the patients with SCAD were on aspirin. The HDs were not taking any drugs and none of the SCAD patients were taking a P2Y12 inhibitor or anticoagulant.

Sample source

Citrate anti-coagulated blood samples (50 mL) were taken from each subject and collected in vacutainers (BD Vacutainer, Becton Dickinson, San Diego, CA) at a final sodium citrate concentration of 3.8%.

Platelet purification

Leukocyte depletion was used to obtain highly purified platelets, as previously described[3,4]. The blood samples were centrifuged at 160 g for 20 minutes at room temperature (RT) in order to obtain platelet-rich plasma (PRP), and the platelets were then purified by immunomagnetic negative selection using magnetic beads coated with anti-CD45 antibodies (DynabeadsH, Invitrogen, Carlsbad, CA) to deplete the nucleated cells. Briefly, PRP was stained with the magnetic bead-coated anti-CD45 monoclonal antibody (mAb) for 20 minutes at RT on a rotator, placed in a magnetic field, and the leukocyte-depleted platelets (LDPs) were than collected as the negative fraction. The purified platelets were washed three times in PBS/BSA solution, counted, and tested for purity by means of anti-CD41 staining and flow cytometry analysis (only samples containing >98% $CD41^+$ cells). Finally, the LDPs were treated with an appropriate amount of TRIzol™ (Invitrogen) for cell lysis and RNA cryopreservation.

Microarray hybridisation

RNA was extracted using TRIzol™ (Invitrogen) in accordance with the manufacturer's protocol, quantified on a Nanodrop ND-100 spectrophotometer, and quality assessed by means of analysis on an Agilent 2200 TapeStation (Agilent Tehnologies, Palo Alto, CA). Fragmented biotin-labelled cDNA (from 10 ng of RNA) was synthesised using the GeneChip WT Pico kit (Affymetrix, Santa Clara, CA). Affymetrix gene chips (Human Transcriptome Array 2.0, Affymetrix, Santa Clara, CA) were hybridised with 5 µg of fragmented and biotin-labelled cDNA in 200 µL of hybridisation cocktail. Target denaturation was performed at 99°C for 5 min. and then at 45°C for 5 min, followed by hybridisation with rotation at 60 rpm for 16 hours at 45°C. The arrays were then washed and stained using a Gene Chip Fluidic Station 450 and an Affymetrix GeneChip hybridisation wash & stain kit. The chips were scanned by an Affymetrix Gene Chip Scanner 3000 using Command Console Software. The experiment was quality controlled using Expression Console Software v 1.4.1.

Microarray data processing

The expression data obtained from the Affymetrix probe-sets (70,523) were processed using Expression Console software (Affymetrix, www.affymetrix.com) with the default parameters. In detail, the procedure applied Signal Space Transformation (SST), which increases the overall absolute-fold changes of HTA2.0 arrays. The data were then Robust Multiarray Average (RMA) background corrected, median polish summarised and quantile normalised. The internal control probe-sets were filtered out, and only the 67,528 probe-sets that map biological features (genes, non-coding genes, etc.) were maintained for downstream analysis. The data are available at GEO, Accession No. GSE109048

Quantitative real-time polymerase chain reaction (PCR)

RNA was extracted using TRIzol™ (Invitrogen) in accordance with the manufacturer's protocol, as previously described[3].

The RNA was reverse-transcribed using the Superscript-III Reverse Transcriptase (Thermo Fisher Scientific, Waltham, MA, USA) and random hexamers (Promega, Madison, WI, USA), in accordance with the manufacturer instructions.

Semi-quantitative real-time RT-PCRs were performed to detect the expression levels of CLEC4E, FKBP5, SAMSN1, S100A12 and S100P using the SYBR Premix Ex Taq II (Takara, Shiga, Japan) and a LightCycler 480 (Roche, Basel, Switzerland). ACTB (Actin Beta) and ITGA2B (Integrin, Alpha 2b, Platelet Glycoprotein IIb of IIb/IIIa Complex, Antigen CD41) were used as housekeeping genes; the reactions were performed in triplicate, and the expression data were analysed and rescaled using GeNorm software[5](Supplemental Figure 3).

The t-test analyses were performed using R software (http://www.r-project.org/).

Statistical analysis

The statistical analysis was based on 38 patients (one patient in the STEMI group and one HD were excluded because of quantitatively low RNA levels). The expression levels of the 10% most variably expressed genes[6] were collected into a 38x6,754 matrix whose rows corresponded to the individual samples and columns corresponded to the selected genes, plus a column for individual STEMI-HD status. Simple logistic regression models[7] were used to test each of the 6,753 expression variables for any association with the STEMI *vs* HD indicator. There were 51 gene expression variables with a p-value of <0.05 (Supplemental Table 1) and five with a p-value of <0.01. The later were considered for the subsequent stage of analysis, in which they acted as explanatory variables in a multiple logistic regression model for the log-odds ratio of the risk of STEMI *vs* HD. The model was internally validated by means of bootstrap resampling[8,9]. We then ran 30,000 iterations of a bootstrap procedure, each iteration involving resampling with the replacement of the rows of the original data matrix in such a way as to preserve the original matched structure, and analysed the simulated dataset using the same procedure as that applied to the original dataset. The 30,000 bootstrapped AUC statistics were used to calculate an empirical mean value and 95% confidence interval for the "true" AUC value. Finally, our logistic predictor of the risk of STEMI *vs* HD was assessed in terms of its ability to discriminate STEMI from SCAD as an external validation of the model. This involved the use of data from further patients with SCAD – the phenotypically closest condition to STEMI with documented coronary atherosclerosis but without thrombosis (~~Supplemental~~ Figure 1, panel B). In this analysis, the logistic predictor was used with the gene-specific coefficients fixed at the values estimated on the basis of the STEMI *vs* HD analysis. The analyses were made using custom R-scripts and R-3.3.2[10].

Reticulated platelet analysis

To exclude the possibility that newly-produced platelets make any significant contribution to DEGs, as it has been reported that acute coronary syndromes are associated with increased platelet turnover during the days following the acute event [11-13], we assessed reticulated platelets frequency in our samples. Briefly, aliquots of whole blood were collected within six hours of the onset of chest pain/symptoms and immediately stained with thiazole orange (TO) and analysed by means of flow cytometry. Briefly, 5 µL of whole blood were diluted in 100 µL of Dulbecco's phosphate buffer (PBS) (Euroclone, Milan, Italy) and incubated with 10 µL mouse anti-human CD41a-PeCy5 monoclonal antibody (Beckton Dickinson, San Diego, CA) for 15 minutes at room temperature in the dark. Then, 400 µL of the TO mix solution (PBS/TO 62.5 ng/mL to obtain a TO staining concentration of 50 ng/mL) (Sigma-Aldrich, St. Louis, MO, USA) or PBS (for the negative control) were added, and incubated for an additional 45 minutes at room temperature in the dark. After incubation, the samples were fixed by adding 500 µL of 2% buffered

paraformaldehyde, and analysed using an Epics XL flow cytometer (Beckman Coulter, Fullerton, CA) with Expo ADC software (Beckman Coulter).

2. Supplemental Figures and Figures legends.

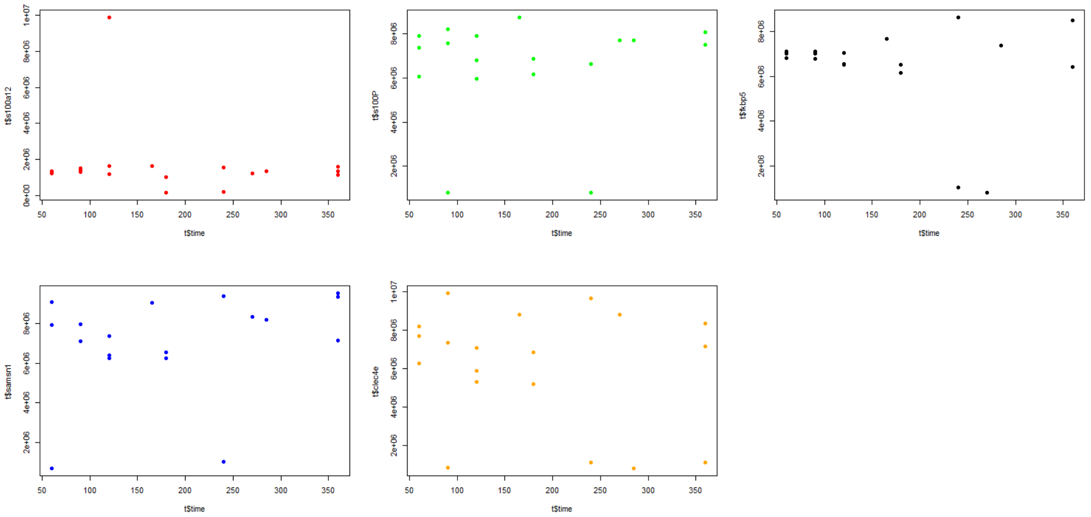

Supplemental Figure 1. Scatterplots of the correlation between time of blood withdrawal (X axis) and gene expression levels (Y axis) for each STEMI patient.

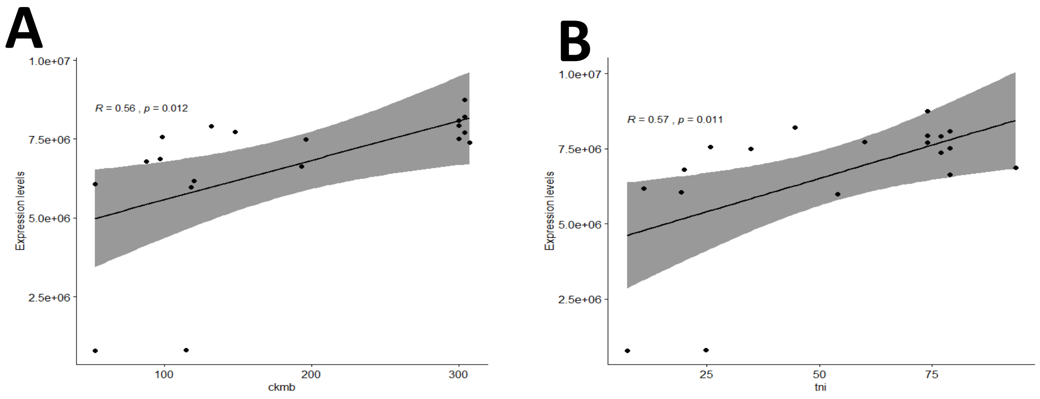

Supplemental Figure 2. Panel A: scatterplots of the correlation between CK-MB levels (X Axis) and S100P expression levels (Y axis). **Panel B:** scatterplots of the correlation between cardiac Troponin-I levels (X Axis) and S100P expression levels (Y axis).

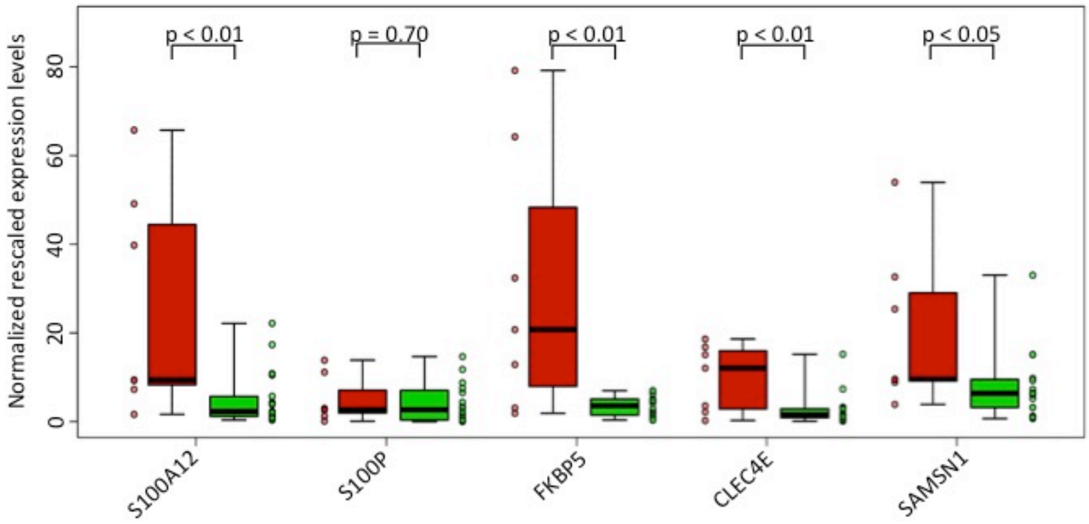

Supplemental Figure 3.

Individual gene expression values as measured by means of real-time RT-PCR in STEMI patients (red) and HD controls (green) with the median value and interquartile range of the five identified genes. The results were normalised to the expression levels of the ACTB and ITGA2B genes, and three technical replicates were performed for each sample. The significance level of t-test analyses is shown.

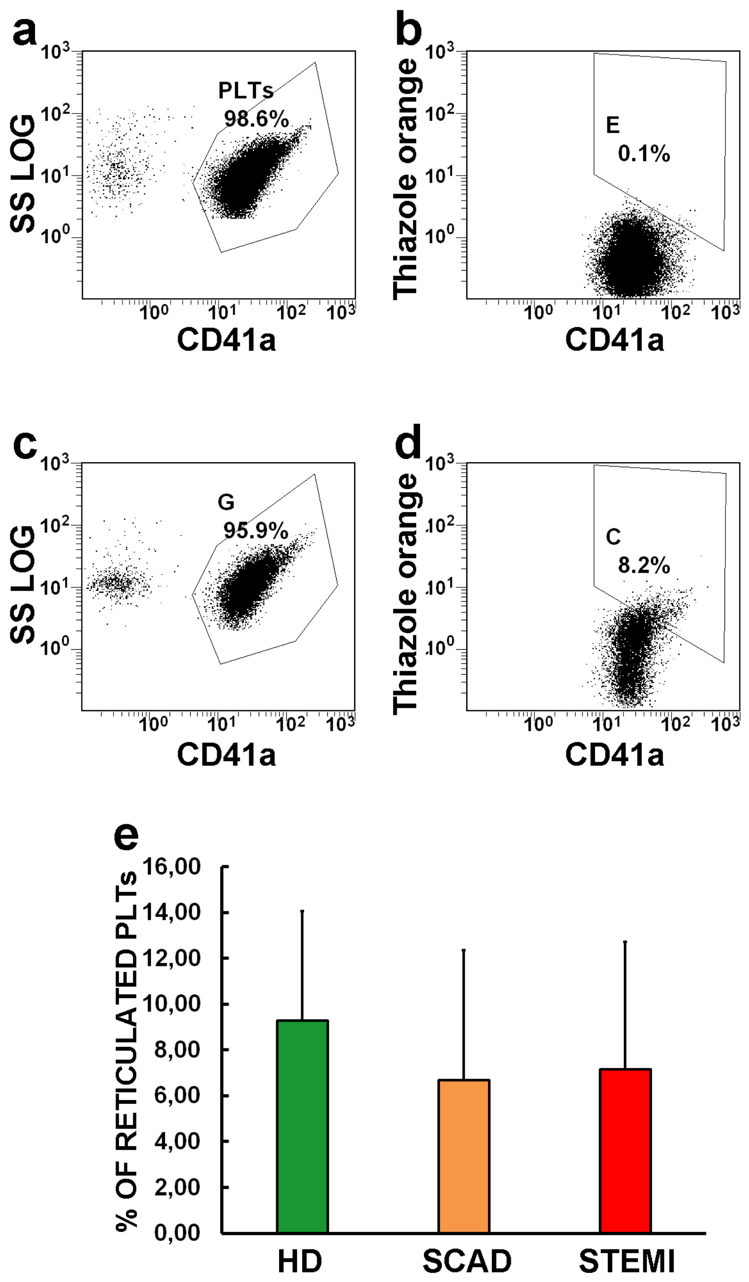

Supplemental Figure 4.

Analysis of reticulated platelets in HDs and SCAD and STEMI patients. a-d: Gating strategy for reticulated platelet analysis. Platelets (PLTs) were identified as CD41a-positive cells (panels a and c), and then analyzed in another dot plot showing CD41a-positive cells vs TO-positive cells in negative controls (panel b) and stained samples (panel d). Reticulated platelets are represented as CD41/TO-positive cells. e: Percentage of reticulated platelets in five HDs, seven SCAD patients and six STEMI patients. Mean values of CD41a/TO-positive platelets ± SD.

Supplemental Table 1. The 51 genes associated with the STEMI vs HD indicator with a p-value of <0.05.

List of identified genes whose expression levels associated with the STEMI *vs* HD indicator with a p-value of <0.05. Bold characters highlight the five genes of the signature (p-value of <0.01).

Probeset	Biomarker	STEMI: HD OR (95% CI)	p-value
**TC01003260.hg.1**	**S100A12**	2.35 (1.51-4.52)	0.0017
**TC06004150.hg.1**	**FKBP5**	204.06 (12.11-15705.95)	0.0026
**TC21001069.hg.1**	**SAMSN1**	2.76 (1.42-6.53)	0.0075
**TC12001178.hg.1**	**CLEC4E**	1.97 (1.26-3.47)	0.0079
**TC04000072.hg.1**	**S100P**	3.35 (1.48-9.25)	0.0082
**TC01001254.hg.1**	S100A9	2.31 (1.29-4.77)	0.0104
**TC13000871.hg.1**	IRS2	5.56 (1.74-25.74)	0.0108
**TC04000856.hg.1**	SAP30	32.29 (3.83-876.22)	0.0123
**TC03000563.hg.1**	TC03000563.hg.1	2.04 (1.24-3.98)	0.0138
**TC01003261.hg.1**	S100A8	3.08 (1.33-8.49)	0.0154
**TC01002372.hg.1**	---	5.41 (1.59-25.27)	0.0155
**TC01000510.hg.1**	SMAP2	2.92 (1.39-8.11)	0.0157
**TC12002425.hg.1**	IRAK3	3.79 (1.48-14.27)	0.0178
**TC16001896.hg.1**	linc-TOX3-4	2.78 (1.26-7.06)	0.0178
**TC19002254.hg.1**	ZNF667-AS1	0.33 (0.12-0.77)	0.0197
**TC01005397.hg.1**	CR597056	2.79 (1.3-7.59)	0.0202
**TC07002399.hg.1**	NCF1B	2.21 (1.17-4.75)	0.024
**TC04000965.hg.1**	---	0.29 (0.09-0.78)	0.0248
**TC13001572.hg.1**	---	4.12 (1.37-16.9)	0.0253
**TC02002528.hg.1**	ENST00000417539	0.53 (0.28-0.89)	0.0296
**TC02003777.hg.1**	uc010fuc.1	0.71 (0.51-0.96)	0.0312
**TC11000824.hg.1**	ACER3	2.23 (1.15-5.26)	0.0318
**TC15001008.hg.1**	ENST00000553658	0.31 (0.09-0.81)	0.0318
**TC09001548.hg.1**	GGTA1P	0.34 (0.11-0.81)	0.0325
**TC13001686.hg.1**	---	0.32 (0.1-0.82)	0.0334
**TC04002623.hg.1**	SPARCL1	2.15 (1.15-4.83)	0.034
**TC19000924.hg.1**	ZNF667-AS1	0.37 (0.13-0.87)	0.0342
**TC11002483.hg.1**	IFITM2	1.93 (1.11-3.89)	0.0346
**TC07000438.hg.1**	NCF1B	2.04 (1.1-4.24)	0.0349
**TC0X002155.hg.1**	BTK	0.48 (0.21-0.88)	0.0354
**TC04002084.hg.1**	TCONS_l2_00020599-	0.43 (0.17-0.84)	0.0365
**TC19002252.hg.1**	ZNF542P	0.34 (0.11-0.85)	0.037
**TC02003367.hg.1**	FUNDC2P2	0.4 (0.15-0.88)	0.0374
**TC09002775.hg.1**	GGTA1P	0.43 (0.17-0.86)	0.0377
**TC06003570.hg.1**	---	5.45 (1.35-34.19)	0.0381
**TC0X001218.hg.1**	BTK	0.37 (0.12-0.86)	0.039
**TC06001488.hg.1**	IFITM4P	4.55 (1.26-23.45)	0.0391
**TC6_mcf_hap5000107.**	IFITM4P	4.55 (1.26-23.45)	0.0391
**TC07001516.hg.1**	NCF1C	2.22 (1.1-5.18)	0.0392
**TC15000268.hg.1**	C15orf54	0.52 (0.24-0.88)	0.0395

**TC09001985.hg.1**	TCONS_l2_00028817	0.73 (0.52-0.96)	0.0406
**TC07003026.hg.1**	NCF1C	2.08 (1.08-4.54)	0.0414
**TC22000381.hg.1**	DQ586951	0.47 (0.21-0.9)	0.0419
**TC06001350.hg.1**	HIST1H2BG	0.69 (0.46-0.95)	0.0424
**TC11001230.hg.1**	IFITM3	2.6 (1.14-7.65)	0.0433
**TC09001372.hg.1**	HSD17B3	0.36 (0.11-0.88)	0.0441
**TC0X002204.hg.1**	SLC25A5	0.6 (0.34-0.96)	0.0463
**TC19000134.hg.1**	MCEMP1	2.4 (1.08-6.3)	0.0467
**TC01000152.hg.1**	TC01000152.hg.1	1.56 (1.03-2.53)	0.0476
**TC19000921.hg.1**	ZNF542P	0.36 (0.11-0.9)	0.0494
**TC08001365.hg.1**	FABP4	3.08 (1.15-11.33)	0.0494

Supplemental Table 2. Clinical, electrocardiographic, and angiographic variables of the STEMI patients.
Family history of ischemic heart disease in a first-degree relative before 55 years in men and before 65 years in women; HT: hypertension; DM: diabetes mellitus; Time: time between symptom onset and blood sampling (the time between hospital admission and blood sampling was always 1:00 hour); Volume: volume of blood sampling; ST elevation: location of ST elevation; CX: circumflex coronary artery; LAD: left anterior descending coronary artery; RCA: right coronary artery; Thrombosis: presence of coronary thrombosis at angiography; CK-MB: CK-MB peak in ng/mL; Tn-I: Troponin I peak in ng/Ml.

Number	Name	Age	Sex	Family history	HT	Smoking	Dyslipidemia	DM	Obesity	Time	Volume	ST elevation	Angiography	Thrombosis	CK-MB	Tn-I	Aspirin	P2Y12 Inhibitors	Anticoagulants
**1**	CL	51	M	YES	YES	NO	YES	NO	NO	1:30 h	50 mL	Inferior-lateral	CX occlusion	YES	115	24,8	YES	NONE	NONE
**2**	PA	54	M	YES	NO	NO	YES	NO	NO	1:30 h	50 mL	Anterior	LAD occlusion	YES	304	44,5	NO	NONE	NONE
**3**	BR	57	M	NO	NO	YES	NO	NO	NO	3:00 h	50 mL	Inferior	RCA occlusion	YES	97,2	93,5	NO	NONE	NONE
**4**	BG	58	M	YES	YES	YES	YES	NO	NO	4:00 h	50 mL	Anterior	LAD occlusion	YES	52,9	7,3	NO	NONE	NONE
**5**	CO	62	M	NO	NO	YES	YES	NO	NO	2:00 h	50 mL	Inferior	RCA occlusion	YES	88	20	NO	NONE	NONE
**6**	PP	65	M	NO	NO	YES	YES	NO	NO	1:30 h	50 mL	Inferior	RCA occlusion	YES	98,6	25,8	NO	NONE	NONE
**7**	RR	70	M	NO	NO	NO	NO	NO	NO	4:00 h	50 mL	Anterior	LAD occlusion	YES	193	> 78	NO	NONE	NONE
**8**	BL	75	M	NO	YES	NO	YES	NO	NO	6:00 h	50 mL	Inferior-lateral	CX occlusion	YES	> 300	> 78	NO	NONE	NONE
**9**	BA	76	M	YES	YES	NO	NO	YES	NO	4:30 h	50 mL	Anterior	LAD occlusion	YES	304	74	NO	NONE	NONE
**10**	BP	70	M	NO	YES	NO	NO	YES	NO	1:00 h	50 mL	Anterior	LAD occlusion	YES	307	77	NO	NONE	NONE
**11**	AA	56	M	YES	NO	YES	NO	NO	NO	1:00 h	50 mL	Inferior	RCA occlusion	YES	53	19,4	NO	NONE	NONE
**12**	CM	69	M	NO	YES	NO	NO	YES	NO	2:00 h	50 mL	Inferior-lateral	CX occlusion	YES	132	77	YES	NONE	NONE
**13**	GG	48	M	YES	YES	YES	YES	YES	YES	1:00 h	50 mL	Anterior	LAD occlusion	YES	304	78	YES	NONE	NONE
**14**	GR	60	M	NO	NO	NO	NO	NO	NO	6:00 h	50 mL	Inferior	RCA occlusion	YES	196	34,8	NO	NONE	NONE

**15**	FE	66	M	NO	YES	YES	NO	NO	NO	2:00 h	50 mL	Lateral	CX occlusion	YES	118	54	YES	NONE	NONE
**16**	AP	51	F	YES	YES	YES	NO	NO	NO	4:45 h	50 mL	Anterior	LAD occlusion	YES	148	60	NO	NONE	NONE
**17**	CE	66	F	NO	NO	YES	NO	NO	NO	3:00 h	50 mL	Anterior	LAD occlusion	YES	120	11	NO	NONE	NONE
**18**	CM	71	F	NO	YES	NO	YES	NO	NO	1:00 h	50 mL	Inferior	RCA occlusion	YES	300	74	YES	NONE	NONE
**19**	GA	82	F	NO	YES	NO	YES	YES	YES	6:00 h	50 mL	Inferior	RCA occlusion	YES	>300	>78	YES	NONE	NONE
**20**	MM	73	F	NO	NO	YES	NO	NO	NO	2:45 h	50 mL	Anterior	LAD occlusion	YES	>304	>74	NO	NONE	NONE

Supplemental Table 3. Clinical, electrocardiographic, and angiographic variables of HDs

Family history of ischemic heart disease in a first-degree relative before 55 years in men and before 65 years in women; HT: hypertension; DM: diabetes mellitus; Time: time between first evaluation and blood sampling; Volume: volume of blood sampling; ST elevation: presence of ST elevation; N/A: not applicable because angiography was not performed; Thrombosis: presence of thrombosis; N/A: not applicable because angiography was not performed; CK-MB: CK-MB level; Tn-I: TnI level.

Number	Name	Age	Sex	Family history	HT	Smoking	Dyslipidemia	DM	Obesity	Time	Volume	ST elevation	Angiography	Thrombosis	CK-MB	Tn-I	Aspirin	P2Y12 Inhibitors	Anticoagulants
1	VM	54	M	NO	NO	NO	NO	NO	NO	1:00 h	50 mL	NO	N/A	N/A	Normal	Normal	NONE	NONE	NONE
2	GG	53	M	NO	NO	NO	NO	NO	NO	1:00 h	50 mL	NO	N/A	N/A	Normal	Normal	NONE	NONE	NONE
3	MM	55	M	NO	NO	NO	NO	NO	NO	1:00 h	50 mL	NO	N/A	N/A	Normal	Normal	NONE	NONE	NONE
4	GS	61	M	NO	NO	NO	NO	NO	NO	1:00 h	50 mL	NO	N/A	N/A	Normal	Normal	NONE	NONE	NONE
5	AD	61	M	NO	NO	NO	NO	NO	NO	1:00 h	50 mL	NO	N/A	N/A	Normal	Normal	NONE	NONE	NONE
6	PA	62	M	NO	NO	NO	NO	NO	NO	1:00 h	50 mL	NO	N/A	N/A	Normal	Normal	NONE	NONE	NONE
7	BV	68	M	NO	NO	NO	NO	NO	NO	1:00 h	50 mL	NO	N/A	N/A	Normal	Normal	NONE	NONE	NONE
8	FL	72	M	NO	NO	NO	NO	NO	NO	1:00 h	50 mL	NO	N/A	N/A	Normal	Normal	NONE	NONE	NONE
9	FR	77	M	NO	NO	NO	NO	NO	NO	1:00 h	50 mL	NO	N/A	N/A	Normal	Normal	NONE	NONE	NONE
10	PE	70	M	NO	NO	NO	NO	NO	NO	1:00 h	50 mL	NO	N/A	N/A	Normal	Normal	NONE	NONE	NONE
11	BG	59	M	NO	NO	NO	NO	NO	NO	1:00 h	50 mL	NO	N/A	N/A	Normal	Normal	NONE	NONE	NONE
12	AA	68	M	NO	NO	NO	NO	NO	NO	1:00 h	50 mL	NO	N/A	N/A	Normal	Normal	NONE	NONE	NONE
13	SM	49	M	NO	NO	NO	NO	NO	NO	1:00 h	50 mL	NO	N/A	N/A	Normal	Normal	NONE	NONE	NONE
14	GA	62	M	NO	NO	NO	NO	NO	NO	1:00 h	50 mL	NO	N/A	N/A	Normal	Normal	NONE	NONE	NONE
15	TU	63	M	NO	NO	NO	NO	NO	NO	1:00 h	50 mL	NO	N/A	N/A	Normal	Normal	NONE	NONE	NONE
16	AA	52	F	NO	NO	NO	NO	NO	NO	1:00 h	50 mL	NO	N/A	N/A	Normal	Normal	NONE	NONE	NONE

**17**	CE	65	F	NO	NO	NO	NO	NO	NO	1:00 h	50 mL	NO	N/A	N/A	Normal	Normal	NONE	NONE	NONE
**18**	RD	70	F	NO	NO	NO	NO	NO	NO	1:00 h	50 mL	NO	N/A	N/A	Normal	Normal	NONE	NONE	NONE
**19**	AM	81	F	NO	NO	NO	NO	NO	NO	1:00 h	50 mL	NO	N/A	N/A	Normal	Normal	NONE	NONE	NONE
**20**	BF	73	F	NO	NO	NO	NO	NO	NO	1:00 h	50 mL	NO	N/A	N/A	Normal	Normal	NONE	NONE	NONE

Supplemental Table 4. Clinical, electrocardiographic, and angiographic variables of SCAD patients.

Family history of ischemic heart disease in a first-degree relative before 55 years in men and before 65 years in women; HT: hypertension; DM: diabetes mellitus; Time: time between first evaluation and blood sampling; Volume: volume of blood sampling; ST elevation: presence of ST elevation; CX: circumflex coronary artery; LAD: left anterior descending coronary artery; RCA: right coronary artery; Thrombosis: presence of coronary thrombosis at angiography; CK-MB; CK-MB level; Tn-I: TnI level.

Number	Name	Age	Sex	Family history	HT	Smoking	Dyslipidemia	DM	Obesity	Time	Volume	ST elevation	Angiography	Thrombosis	CK-MB	Tn-I	Aspirin	P2Y12 Inhibitors	Anticoagulants
1	MM	55	M	YES	YES	NO	YES	YES	NO	1:00 h	50 mL	NO	RCA, LAD	NO	Normal	Normal	YES	NONE	NONE
2	MD	56	M	NO	YES	NO	NO	NO	NO	1:00 h	50 mL	NO	CX, LAD, RCA	NO	Normal	Normal	YES	NONE	NONE
3	FR	57	M	NO	YES	NO	YES	NO	YES	1:00 h	50 mL	NO	LAD	NO	Normal	Normal	YES	NONE	NONE
4	SA	59	M	NO	YES	YES	YES	NO	NO	1:00 h	50 mL	NO	LAD	NO	Normal	Normal	YES	NONE	NONE
5	OC	61	M	NO	YES	NO	YES	NO	NO	1:00 h	50 mL	NO	CX, RCA	NO	Normal	Normal	YES	NONE	NONE
6	GM	62	M	NO	NO	NO	NO	YES	NO	1:00 h	50 mL	NO	LAD	NO	Normal	Normal	YES	NONE	NONE
7	AG	67	M	NO	YES	NO	NO	NO	NO	1:00 h	50 mL	NO	LAD, CX	NO	Normal	Normal	YES	NONE	NONE
8	SR	74	M	YES	YES	NO	YES	NO	NO	1:00 h	50 mL	NO	LAD, CX	NO	Normal	Normal	YES	NONE	NONE
9	BR	74	M	NO	YES	NO	NO	NO	NO	1:00 h	50 mL	NO	LAD	NO	Normal	Normal	YES	NONE	NONE
10	AG	72	M	NO	YES	NO	YES	NO	YES	1:00 h	50 mL	NO	CX	NO	Normal	Normal	YES	NONE	NONE
11	CG	58	M	NO	NO	NO	NO	YES	NO	1:00 h	50 mL	NO	RCA	NO	Normal	Normal	YES	NONE	NONE
12	MM	68	M	NO	YES	NO	NO	NO	NO	1:00 h	50 mL	NO	LAD	NO	Normal	Normal	YES	NONE	NONE
13	GG	51	M	NO	YES	NO	NO	NO	NO	1:00 h	50 mL	NO	LAD	NO	Normal	Normal	YES	NONE	NONE
14	DS	64	M	YES	YES	YES	YES	YES	NO	1:00 h	50 mL	NO	LAD, RCA, CX	NO	Normal	Normal	YES	NONE	NONE
15	IP	66	M	NO	YES	NO	NO	YES	NO	1:00 h	50 mL	NO	LAD	NO	Normal	Normal	YES	NONE	NONE
16	VD	54	F	NO	NO	YES	NO	NO	NO	1:00 h	50 mL	NO	LAD	NO	Normal	Normal	YES	NONE	NONE

**17**	BM	63	F	NO	YES	NO	YES	YES	NO	1:00 h	50 mL	NO	LAD	NO	Normal	Normal	YES	NONE	NONE
**18**	SA	72	F	NO	YES	NO	YES	NO	YES	1:00 h	50 mL	NO	LAD, RCA, CX	NO	Normal	Normal	YES	NONE	NONE
**19**	DM	80	F	NO	YES	NO	YES	YES	NO	1:00 h	50 mL	NO	LAD, RCA, CX	NO	Normal	Normal	YES	NONE	NONE
**20**	MM	75	F	NO	YES	NO	YES	NO	NO	1:00 h	50 mL	NO	LAD	NO	Normal	Normal	YES	NONE	NONE

Supporting Information

The expression of the 5 DEGs were also investigated by qRT-PCR, confirming the up-regulation of FKBP5, SAMSN1, S100A12 and CLEC4E, in STEMI patients as compared to HD. (Supplemental Figure 3).

As it has been reported that acute coronary syndromes are associated with increased platelet turnover during the days following the acute event [11-13], we assessed reticulated platelets frequency in our samples and found that it was similar in all of the groups (STEMI: 7.15±5.58%; SCAD: 6.69±5.68%; HD: 9.28±4.76%) (Supplemental Figure 4), thus excluding the possibility that newly-produced platelets make any significant contribution to DEGs.

4. Supplemental References.

1. Thygesen, K., Alpert, J.S., Jaffe, A.S., Simoons, M.L., Chaitman, B.R. & White, H.D. The Writing Group on behalf of the Joint ESC/ACCF/AHA/WHF Task Force for the Universal Definition of Myocardial Infarction. Third universal definition of myocardial infarction. *Circulation.* **126,** 2020-2035 (2012).
2. Montalescot, G., Sechtem, U. & Achenbach, S. 2013 ESC guidelines on the management of stable coronary artery disease. *European. Heart. Journal*. **34,** 2949–3003 (2013).
3. Carubbi, C., et al. Protein kinase C ε expression in platelets from patients with acute myocardial infarction. *PLoS One.* 7:e46409 (2012).
4. Carubbi, C., et al. Cytofluorimetric platelet analysis. *Semin. Thromb. Hemost.* **40,** 88-98 (2014).
5. Vandesompele, J., et al. Accurate normalization of real-time quantitative RT-PCR data by geometric averaging of multiple internal control genes. *Genome. Biol.* **3,** RESEARCH0034 (2002).
6. Bourgon, R., Gentleman, R. & Huber W. Independent filtering increases detection power for high-throughput experiments. *Proc. Natl. Acad. Sci. U S A.* **107,** 9546-9551 (2010).
7. Liao, J.G. & Chin KV. Logistic regression for disease classification using microarray data: model selection in a large p and small n case. *Bioinformatics.* **23,**1945-1951 (2007).
8. Efron B. Better bootstrap confidence intervals, *J. Am. Stat. Assoc.* **82,** 171-200 (1987).
9. Berrar, D. & Flach, P. Caveats and pitfalls of ROC analysis in clinical microarray research (and how to avoid them). *Brief Bioinform.* **13,** 83-97 (2012).
10. R Core Team (2017). R: A language and environment for statistical computing. R Foundation for Statistical Computing, Vienna, Austria. URL https://www.R-project.org/.
11. Gonzalez-Porras, J.R., et al. The role of immature platelet fraction in acute coronary syndrome. *Thromb. Haemost*. **103,** 247-249 (2010).
12. Cesari, F., et al. Reticulated platelets predict cardiovascular death in acute coronary syndrome patients. Insights from the AMI-Florence 2 Study. *Thromb. Haemost.* **109,** 846-853 (2013).
13. Hoffmann, J.J. Reticulated platelets: analytical aspects and clinical utility. *Clin. Chem. Lab. Med.* **52,** 1107-1117 (2014).
